# Cellular and animal models for high-throughput screening of therapeutic agents for the treatment of the diseases of the elderly in general and Alzheimer’s disease in particular^†^

**DOI:** 10.3389/fphar.2013.00059

**Published:** 2013-05-13

**Authors:** Jordan L. Holtzman

**Affiliations:** ^1^Department of Pharmacology, University of MinnesotaMinneapolis, MN, USA; ^2^Department of Medicine, University of MinnesotaMinneapolis, MN, USA; ^3^Department of Environmental Health Sciences, University of MinnesotaMinneapolis, MN, USA

**Keywords:** amyloid-β, protein processing, chaperones, *N*-glycosylation, dementia

## Abstract

It is currently thought that the dementia of Alzheimer’s disease is due to the neurotoxicity of the deposits or aggregates of amyloid-β (Aβ) in the extracellular space of the cerebral cortex. This model has been widely criticized because there is a poor correlation between deposits and dementia. Others have questioned whether Aβ is truly neurotoxic. Yet, in spite of these concerns, the search for therapeutic agents has been based on the development of mouse models transfected with mutant genes associated in humans with early onset Alzheimer’s disease. A major limitation of these models is that although they exhibit many of the pathological and clinical manifestation of the human disease, the bulk of individuals who develop the dementia of Alzheimer’s disease have none of these mutant genes. Furthermore, nine clinical trials of drugs that were effective in transgenic mice failed to show any benefit in patients. Finally, a major unresolved issue with the Aβ model is that since Aβ is produced in everyone, why are deposits only seen in the elderly? This issue must be resolved if we are to understand the etiology of the disease and develop test systems for both diagnosis and drug discovery. Published studies from my laboratory demonstrate that in human cerebrospinal fluid immunoreactive Aβ is only present as a complex with two chaperones, ERp57 and calreticulin and is *N*-glycosylated. This complex formation is catalyzed by the posttranslational protein processing system of the endoplasmic reticulum (ER). Others have reported that in plaque Aβ is present only as the naked peptide. Together these results suggest that both plaque and dementia are secondary to an age related decline in the capacity of the ER to catalyze protein, posttranslational processing. Since the synaptic membrane proteins necessary for a functioning memory are also processed in the ER, these findings would suggest that the loss of cognition is due to a decline in the capacity of the neuron to produce and maintain functioning synapses. Work from my laboratory and from others further indicate that the components of the ER, posttranslational, protein processing pathway do dramatically decline with age. These data suggest that this decline may be found in all cells and could account not only for the dementia of Alzheimer’s disease, but also for many of the other manifestations of the aging process. These observations also suggest that declining ER function has a role in two well-recognized phenomena associated with aging: a loss of mitochondrial function and a decrease in myelin. Finally, based on this paradigm I propose new cellular and animals models for high-throughput screening for drug discovery.

## INTRODUCTION

It is currently thought that the dementia of Alzheimer’s disease is due to the neurotoxicity of the deposits or aggregates of amyloid-β (Aβ) in the extracellular space of the cerebral cortex. In support of this model various familial forms of the disease are characterized by the early deposition of plaque and the development of dementia. In light of the similar pathology observed between the familial forms and the sporadic disease, many workers have developed transgenic mouse models in which the animals were transfected with mutant forms of the amyloid precursor protein (APP) and/or presenilins 1 and 2. The APP normally is cleaved to form two growth factors, αsAPP and βsAPP while the presenilins are components of the γ secretase which clips the Aβ from the membrane. The αsAPP is fifty times more active than βsAPP’s (Herzog et al., 2004), but the latter is present at much higher concentrations in the brain (De Strooper et al., 2010).

Yet, a major philosophical problem with this approach is that the vast majority of the elderly with late onset disease do not have these mutations. In fact in genome wide association studies the most consistently observed genetic variants associated with the late onset disease are found in the apoE and clusterin genes. Furthermore, another difference between the late onset and familial forms is that in human studies Pottier et al. (2012) reported that the mRNA for APP was not elevated in late onset Alzheimer’s disease, but was significantly higher in individuals with the various forms of the familial disease and Down’s syndrome. Although western blotting of APP would be more definitive, when increases are seen in mRNAs they are commonly accepted as a measure of the total protein content. Hence, these data might suggest that one possible underlying biochemical defect seen in the familial forms of the disease may result in part from elevated levels of APP. Studies on the role of APP in cellular metabolism may give a clue as to how such elevations might lead to the early onset disease.

Knockout studies of APP have reported that this growth factor is necessary for normal neuronal development (Dawson et al., 1999). Furthermore, studies by Nikolaev et al. (2009) have suggested that APP may play an important role at one particular stage in normal embryonic development. As the brain develops the embryo accumulates an excess of neurons and axons. These investigators reported that APP activates death receptor 6 which leads to axon pruning and apoptosis of the excess neurons. These data might suggest that the loss of cognition seen early in the familial forms of the disease before there is overt neuropathology could result from excessive axon pruning due to the high levels of APP; while later in the progression of the disease the high levels of APP could lead to increased neuronal apoptosis with the development of overt neurohistopathology.

Of greater concern there have been nine phase III clinical trials of agents that were effective in transgenic mice but failed to show any efficacy in patients with late onset disease (**Table 1**).

**Table 1:** Failed phase III trials of agents which were effective in transgenic mouse models.

	Drug	Mechanism	Reference	Animal reference
1	Abeta vaccine	Clear abeta	Holmes et al. (2008)	Janus et al. (2000) and Morgan et al. (2000)
2	Tarenflurbil	γ-Secretase inhibitor	Green et al. (2009)	Kukar et al. (2007)
3	Semagacestat	γ-Secretase inhibitor	Schor (2011)	Abramowski et al. (2008)
4	MK-677	IGF-1 secretagogue	Sevigny et al. (2008)	Carro et al. (2002)
5	*Ginkgo biloba*	Antioxidant	DeKosky et al. (2008) and Vellas et al. (2012)	Stackman et al. (2003)
6	Estrogen	Hormone replacement	Espeland et al. (2004)	Levin-Allerhand et al. (2002) and Carroll et al. (2007)
7	Docosahexaenoic acid	Omega-3 fatty acid	Quinn et al. (2010)	Calon et al. (2004)
8	Bapineuzumab	Monoclonal antibody	Scheltens et al. (2012)	Imbimbo et al. (2012)
9	Solanezumab	Monoclonal antibody	Doody (2012)	Imbimbo et al. (2012)

The probability of nine failures in nine trials for a presumed association is 2^9^ or 1 in 512 which gives a *p* = 0.0195.

Many have ascribed these failures to the initiation of therapy only after the subjects have exhibited significant cognitive decline. This was not true in two of these trials: the GEM (Ginkgo Evaluation of Memory) trial of *Ginkgo biloba* (DeKosky et al., 2008) and the Women’s Health Initiative Trial of estrogen alone (Espeland et al., 2004).

In the GEM trial of *G. biloba* the subjects were followed for 7 years (DeKosky et al., 2008). The investigators observed no difference in the incidence of dementia between the placebo and the *Ginkgo* treated subjects even though this agent was effective in transgenic mice (**Figure 2**; Stackman et al., 2003).

Similarly, in the estrogen only arm of the Women’s Health Initiative Trial the subjects were also followed for 7 years (**Figure 3**; Espeland et al., 2004). After an initial improvement in cognitive scores which was probably due to a training effect, there were similar declines in the scores of the placebo and estrogen groups.

Furthermore, permanent damage to neurons, such as apoptosis and tangle deposits, is thought to only occur late in the disease. Hence, the late administration of effective agents should at least slow the cognitive decline, if not reverse the disease process. In fact in many of the transgenic mouse studies, elderly animals showed cognitive improvement when given some of these agents (Imbimbo et al., 2012).

In all of these trials only solanezumab gave even the faintest hope of slowing this decline. But even this agent failed to meet the primary end points, and the noted benefit was only seen after combining the results of subjects with early disease in the two trials of the drug, suggesting that the effect may not have been sufficiently robust to be of clinical significance (Doody, 2012). The Food and Drug Administration (FDA) has refused to accept these findings as part of a future New Drug Application (NDA).

**FIGURE 1:**
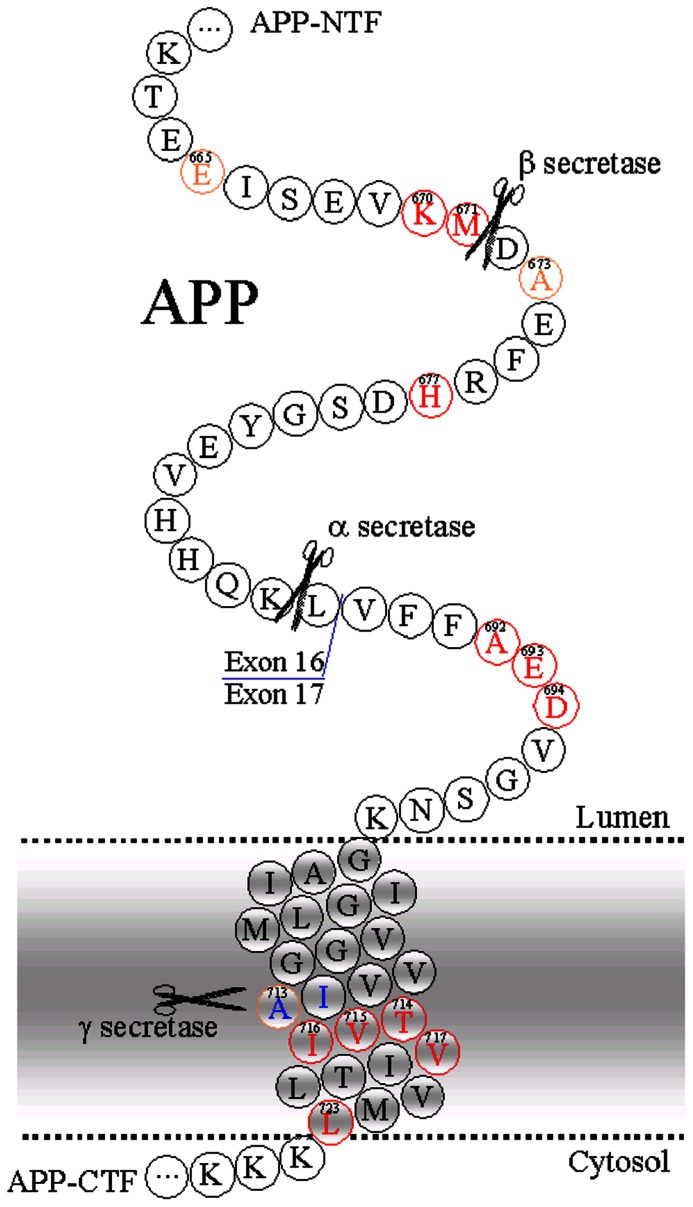
**Amino acid sequence of APP**.

In spite of these failures the general thrust of drug development continues to seek new agents which will decrease the production of Aβ or enhance its clearance. These agents include new antibodies, vaccines, and γ-secretase inhibitors. Also a number of firms have developed inhibitors of the β-secretase (BACE1). This enzyme cleaves APP at the site which yields the Aβ peptide and the βsAPP (**Figure 1**). This would seem to be a somewhat hazardous approach for the long term treatment of patients with cognitive impairment, since knocking out BACE1 has been shown to lead to significant deficits in brain function (Laird et al., 2005; Hu et al., 2010; Rajapaksha et al., 2011). The most serious of these problems is that these animals have been reported to develop a seizure disorder associated with an increased density of surface Na_x_1.2 channels and increased intrinsic firing of isolated neurons and in hippocampal brain slices (Hu et al., 2010). In a short term phase I trial in normal volunteers the Merck BACE1 inhibitor, showed no adverse effects. But considering that these adverse effects may require long term administration, since it may dependent upon inducing the Na_x_1.2 channels, it may take months to observe this toxicity. Furthermore decreasing the level of Aβ in the brain may require higher doses than those used in the phase I trial. Hence, at higher doses for longer treatment periods there could be an increased risk for observing the toxic side effects.

**FIGURE 2:**
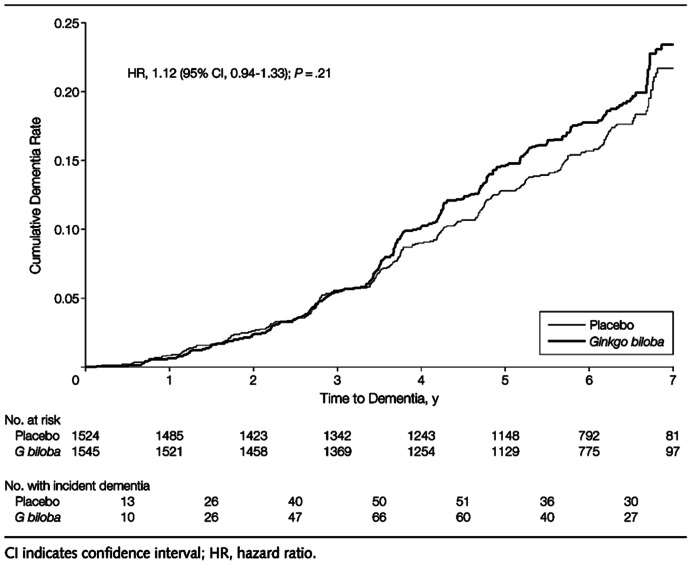
**The effect of *Ginkgo biloba* on Cognitive Function in the GEM Trial (taken from DeKosky et al., 2008)**.

**FIGURE 3:**
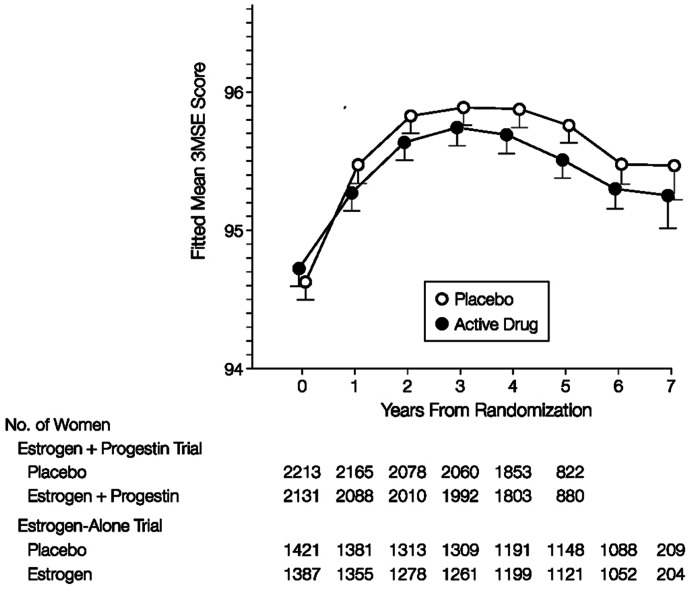
**The effect of estrogen alone on Cognitive Function in the Women’s Health Initiative (taken from Espeland et al., 2004)**.

The only agent which has actually shown any promise of a viable treatment for Alzheimer’s disease is methylene blue (Wischik, 2012). This drug has been used as a urinary tract disinfectant and analgesic since the 1930s. Basic studies have demonstrated that its administration can lead to the loss of tangles, another pathological manifestation of Alzheimer’s disease. Tangles result from cellular stress which leads to hyperphosphorylation of a neuronal, cytoskeletal protein, *tau* (P-*tau*). The P-*tau* then aggregates to form tangles. In a small phase II trial patients receiving 138 mg per day of the drug showed a markedly reduced cognitive decline during a 1 year study period. Yet, those receiving twice the dose showed a parallel decline to the placebo. A chemical modification of this agent is currently going into a phase III clinical trial. It will be interesting to see whether they can replicate their initial findings.

## A NEW PARADIGM FOR THE ETIOLOGY OF THE DEMENTIA OF ALZHEIMER’S DISEASE

In light of these failures it would seem reasonable to examine new paradigms for the etiology of this dreaded condition. A number of years ago we asked the question:

Since Aβ is produced in everyone why is plaque only seen in the brains of the elderly?

**FIGURE 4:**
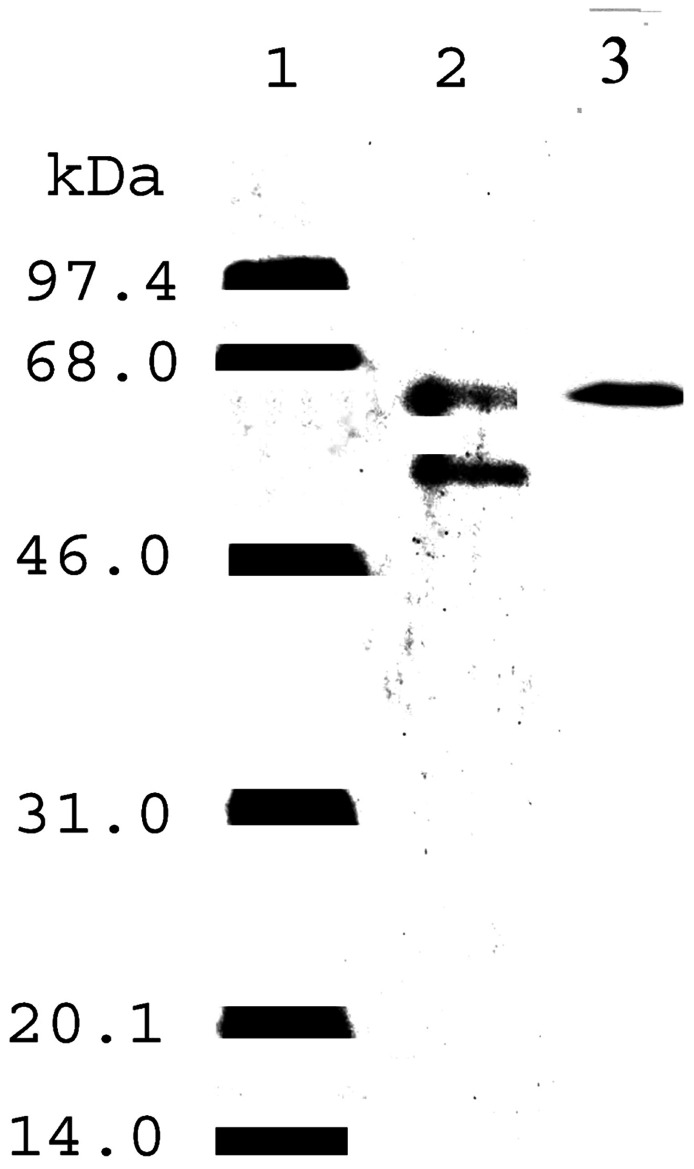
**Western blots of human, CSF with antibodies to ERp57 and Aβ**.

We postulated that normally Aβ is bound to proteins which keep it in solution and thereby prevent plaque formation. And indeed this turned out to be the case! When we ran western blots of fresh or fresh frozen cerebrospinal fluid (CSF) on denaturing gels, we observed that there was only a single Aβ-immunoreactive band which was consistently found at around 62 kDa (**Figure 4**; Erickson et al., 2005). And was associated with an endoplasmic reticulum (ER) chaperone, ERp57.

**FIGURE 5:**
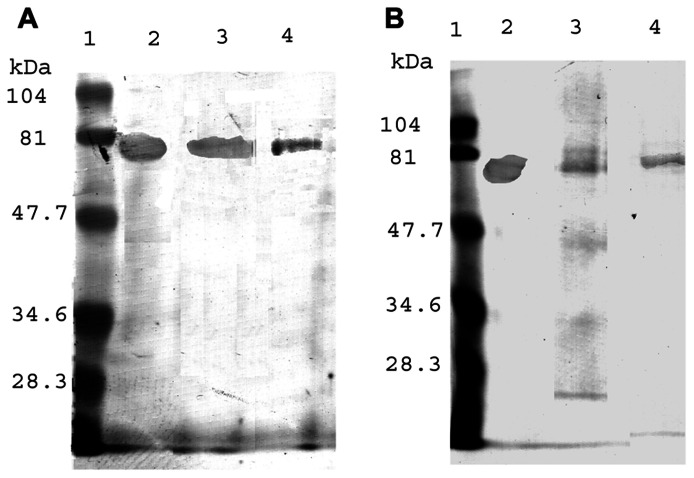
**Immunoopurification of the Aβ**-ERp57 Complex from Human CSF. Aβ and ERp57 were isolated by immunoprecipitation. The samples were then purified by western blotting on sodium docecyl sulfate-polylacrylamide gel electrophoresis. The bands were identified by antibodies to ERp57 **(A)** and Aβ **(B)**. (Taken from Erickson et al., 2005)

Furthermore, on immunopurification we were able to isolate the complex with antibodies to either Aβ or ERp57 (**Figure 5**).

**FIGURE 6:**
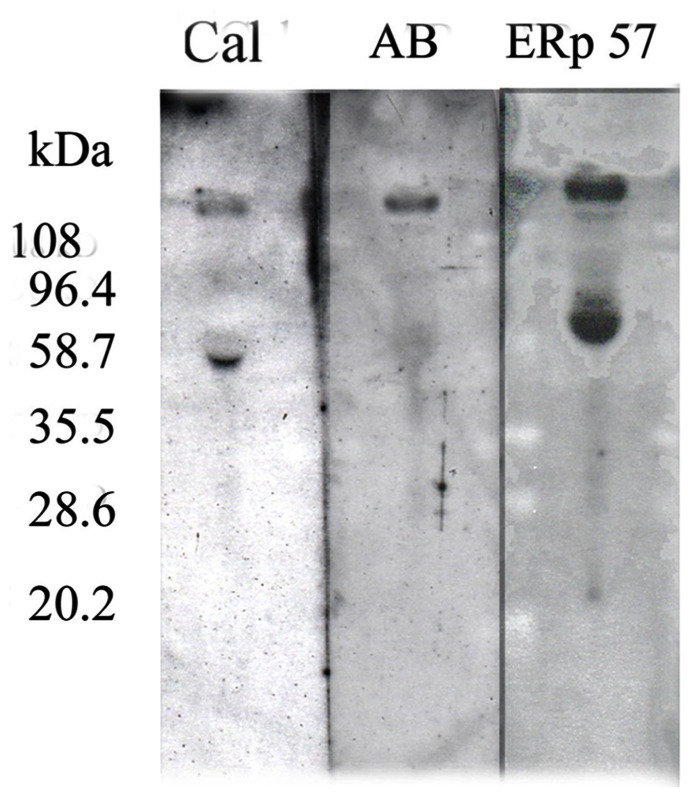
**Western blot of the Aβ–ERp57–calreticulin complex from human CSF after separation on a native gel (taken from Erickson et al., 2005**).

A large number of studies on the biochemistry of the ER, posttranslational, protein processing pathway have reported that ERp57 binds to nascent proteins at *N*-glycosylation sites in association with another ER chaperone, calreticulin. In line with these observations, when the CSF was run on a native gel, the band moved to 118 kDa and was associated with both ERp57 and calreticulin (**Figure 6**).

The fact that the Aβ and the two chaperones all shifted to this higher molecular weight along with the immunopurification data strongly supports our claim that the Aβ band is not either of the two sAPP’s which have similar molecular weights.

**FIGURE 7:**
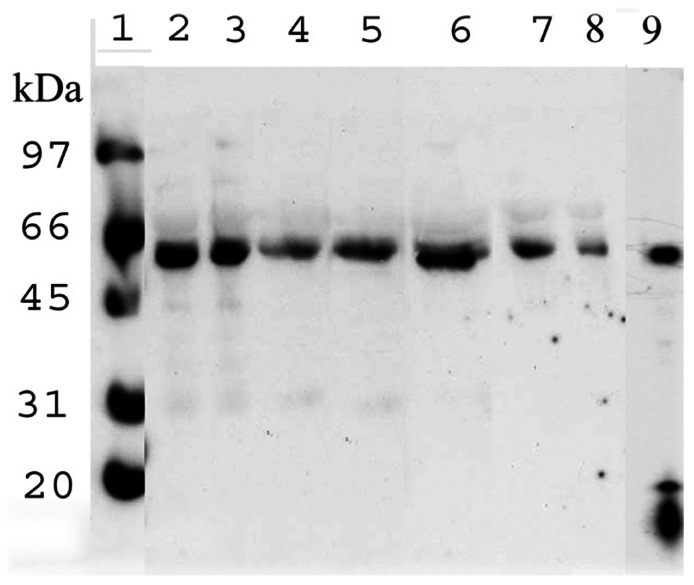
**Immunoblot of Aβ** in CSF after treatments to dissociate the Aβ–ERp57 complex. Channel 1 – molecular weight markers; channel 2 – untreated CSF; channel 3 – CSF treated with 70% formic acid; channel 4 – CSF treated with 80% trifluoroacetic acid; channel 5 – CSF treated with 6 M guanidine isothiocyante; channel 6 – CSF treated with 6 M urea; channel 7 – CSF proteins which did not bind to a boronate column; channel 8 – CSF proteins which bound to a boronate column; channel 9 – treatment of the complex with glycine buffer, pH 9.0 (taken from Erickson et al., 2005).

Finally, since the Aβ found in plaque is only present as the naked peptide, one possibility is that the complex was originally in the plaque but broke down when it was solubilized by the usual treatment with either concentrated formic or trifluoroacetic acid. This is not likely to be the case since treatment of CSF under these same conditions had no effect on the complex (channels 3 and 4) (**Figure 7**).

Chaotropic agents such as urea and guanidine isothio- cyanate also had no effect on the complex (channel 5 and 6). In fact the only procedure which did affect the complex was storage overnight at 4°C in a pH 9.0 buffer (channel 9). The other interesting aspect of this study was that the complex bound to a bonate column, a synthetic lectin, suggesting that the Aβ is glycosylated (channel 8).

The binding of ERp57 to APP is not unique. Two plasma membrane receptors, the vasopressin receptor (Aiyar et al., 1989) and the 1,25 dihydroxy vitamin D plasma membrane receptor (Nemere et al., 2004; Nemere, 2005), have also been shown to have bound ERp57. The latter receptor is present in the plasma membrane of enterocytes and modulates Ca^++^ uptake from the intestines. The ERp57 would appear to have a role in signal transduction since knocking it down or inhibiting it with antibody to its terminal carboxy end inhibited 1,25 dihydroxy vitamin D stimulated Ca^++^ uptake (Nemere et al., 2004; Nemere, 2005). It should also be noted that the title of the publication by Aiyar et al. (1989) demonstrating the binding of ERp57 to the vasopressin receptor is incorrect. When these workers originally cloned and sequenced ERp57, they erroneously identified it as a phosphatidylinositol specific phospholipase C (Bennett et al., 1988). We found that the ERp57 purified from rat liver (Srivastava et al., 1991) and rERp57 generated using the authors’ plasmid had no phospholipase activity (Srivastava et al., 1993) and instead it is a thiol:protein disulfide oxidoreductase: a family of enzymes that catalyzes both the oxidation and reduction of protein sulfur groups and is usually referred to as a Protein Disulfide Isomerase (PDI).

## THE EFFECT OF AGE ON THE COMPONENTS OF THE ER, POSTTRANSLATIONAL, PROTEIN PROCESSING PATHWAY

**FIGURE 8:**
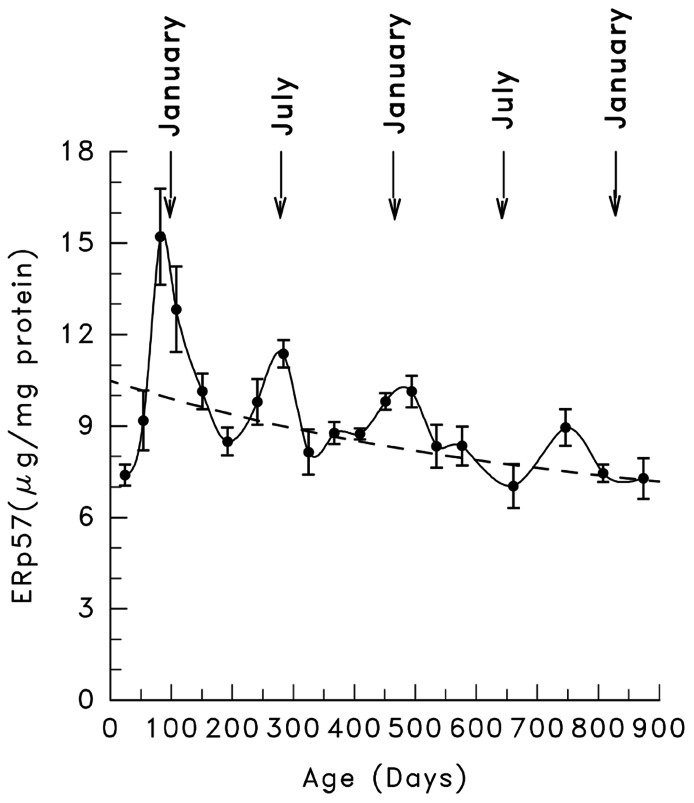
**The effect of age on the ERp57 content of rat, hepatic microsomes (taken from Erickson et al., 2006**).

In light of these findings we hypothesized that the dementia seen in late onset Alzheimer’s disease is due to a decline in the capacity of the neurons to catalyze the ER, posttranslational, protein processing of the synaptic membrane proteins that are necessary for a functioning memory (Erickson et al., 2005)! In support of this hypothesis we found that ERp57 does decline with age (**Figure 8**; Erickson et al., 2006).

Several other ER chaperones showed similar declines (**Table 2**).

**Table 2:** Effect of age on the content of rat liver ER chaperones (taken from Erickson et al., 2006).

Chaperone	Peak concentration μg/mg protein	Concentration @ 874 days μg/mg protein	Constitutive decline %	Show cyclic variation	Cyclic variation % decline
BiP	80.0	48.5	39	Yes	50
Calnexin	57.4	40.5	29	No	–
Calreticulin	7.6	4.8	8	No	–
ERp55	34.8	13.1	51	Yes	73
ERp57	15.4	8.2	32	Yes	71
ERp72	141	100	30	No	–
Total	336.2	215.1	37

Finally, data from other laboratories would suggest that there is also a decline in the *N*-glycosylation pathway with age. Firstly, there is a reduced content of *N*-glycosylated proteins in the tissues of the elderly (Kousvelari et al., 1988). Yet, this decline could be due to decreases in gene transcription. On the other hand, a number of studies have suggested that it may also be due to changes in the activity of the *N*-glycosylation pathway. This concept is based on the effect of age on the content of dolichol in various tissues, a necessary cofactor for *N*-glycosylation.

**FIGURE 9:**
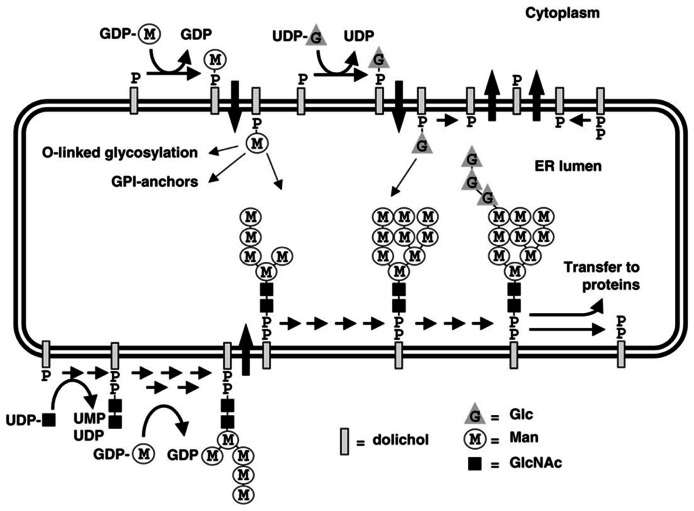
**The *N*-glycosylation pathway (Helenius and Aebi, 2002)**.

The initial step in the *N*-glycosylation pathway is the synthesis of an oligosaccharide bound to dolichol phosphate (Yan and Lennarz, 1999; Helenius and Aebi, 2002; Kelleher and Gilmore, 2006; **Figure 9**). Dolichol is a high molecular weight terpine which serves as a cofactor for the cytosolic synthesis of the oligosaccharide. Once the complex is fully synthesized, it is transferred to the lumen of the ER and attached to an ξ-amino group of an asparagine by an oligosaccharide transferase (OST; Kelleher and Gilmore, 2006). The addition of the individual sugars to the carbohydrate complex is catalyzed by a family of specific monosaccharide transferases (MST; Yan and Lennarz, 1999; Helenius and Aebi, 2002; Kelleher and Gilmore, 2006). The first sugars added to the dolichol phosphate are a pair of *N*-acetylglucosamines, followed by nine mannoses and three glucoses. A large number of studies have shown that as animals age there is a 5–10-fold increase in the cellular content of both dolichol and dolichol phosphate (for example see Marino et al., 1998). Since there is no increase in the synthesis of dolichol, this finding is consistent with a blockage of the addition of the first *N*-acetylglucosamine. And indeed it has been reported that the activity of ALG7, the MST that adds the first N-acetylglucosamine to the dolichol phosphate, does decline with age (Mota et al., 1994).

## OUR STUDIES INDICATE THAT

1. The Aβ-chaperone complex is formed during the normal processing of APP in the lumen of the ER.
2. If this complex is not produced, then the naked peptide is secreted and precipitates on its release from the cell to form plaque.
3. On western blotting of a native gel we found that the complex contains two ER chaperones, ERp57 and calreticulin.
4. In the CSF Aβ is probably also *N*-glycosylated.
5. These data indicate that plaque formation is due to a decline in the capacity of the ER to catalyze the posttranslational processing of APP. It may also account for many other manifestations of aging.
6. Forty percentage of the proteins synthesized in the cell undergo this same posttranslational processing in the ER, including all the synaptic membrane proteins that are necessary for a functioning memory.
7. With age there is a decrease in the components of the ER, protein, posttranslational processing pathway.

## THE ROLE OF DECREASED ER, PROTEIN, POSTTRANSLATIONAL PROCESSING IN THE DECLINE WITH AGE IN MITOCHONDRIAL FUNCTION AND MYELIN CONTENT

There are numerous phenomena associated with the aging process. Two of the most fundamental are the decline with age in mitochondrial function and myelin content. One of the major problems with the Aβ model is that it does not address what the relationship of either of these two well-documented processes has to do with plaque deposition. I feel that both of these processes may be related to the decline in ER function.

### THE ROLE OF THE LOSS OF ER FUNCTION IN THE DECLINE IN MITOCHONDRIAL FUNCTION

It is well-known that as mitochondria age they acquire structural defects (Batlevi and La Spada, 2011). The damaged mitochondria are cleared from the cell by autophagy. Many of the proteins which make up the autophagic vacuoles are processed in the ER and *golgi* (Militello and Colombo, 2011). Hence, with a decline in ER function there is also a decline in autophagy. Such declines have been associated with the onset of some age related neurodegenerative diseases (Pan et al., 2008).

Second, the mitochondria are directly bound to the ER through the mitochondrial associated membrane (MAM; Merkwirth and Langer, 2008; Hayashi et al., 2009; Schon and Przedborski, 2011). This structure serves three, major functions. The first is to transport critical phospholipids from the ER, where they are synthesized, into the mitochondria where they serve vital roles in the maintenance of the structure and function of the mitochondria. Further, it regulates Ca^++^ homeostasis in the mitochondria and thereby oxidative phosphorylation.

**FIGURE 10:**
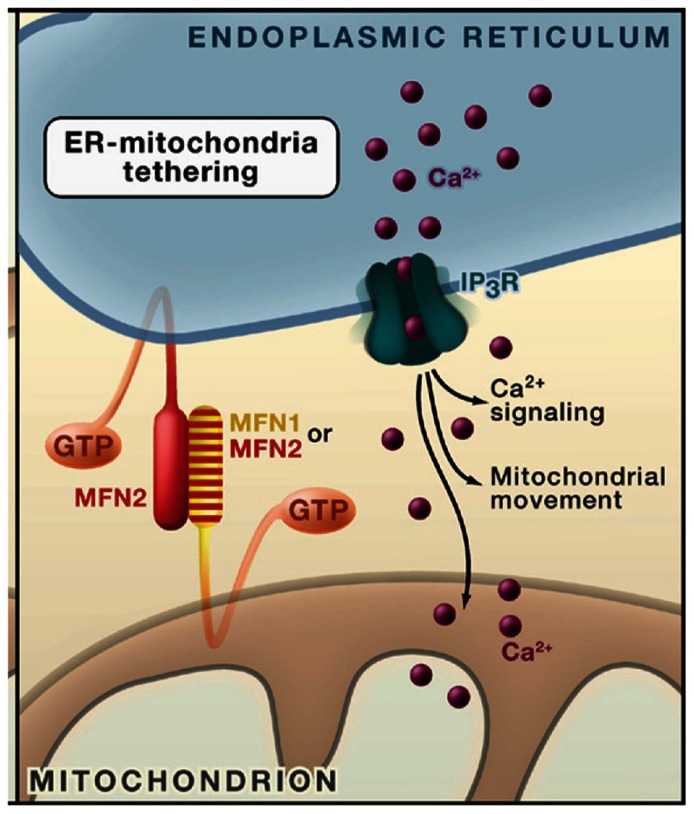
**The tethering of the ER to the mitochondria (taken from Merkwirth and Langer, 2008)**.

A final role for the ER in maintaining mitochondrial structure and function is the control of mitochondrial fission and fusion. As defective mitochondria are cleared by autophagy, they are replaced through a process involving both mitochondrial fission and fusion. Recent studies have indicated that several proteins in the MAM play major roles in both of these processes (Merkwirth and Langer, 2008; Friedman et al., 2011). In particular two critical proteins which tether the ER to the mitochondrial outer membrane are mitofusin 1 and 2 (*mfn1* and *2*; Merkwirth and Langer, 2008; **Figure 10**). These transmembrane proteins are located in both the ER membrane and the outer membrane of the mitochondria. Both *mfn1* and* 2* undergo posttranslational processing in the ER. Hence, a decline in the capacity of the ER to catalyze their posttranslational processing would be expected to have a profound effect on mitochondrial structure and function. Such declines in ER function could compromise the ability of the cell to maintain metabolically active mitochondria.

### THE ROLE OF THE LOSS OF ER FUNCTION IN THE DECREASE IN MYELIN WITH AGE

**FIGURE 11:**
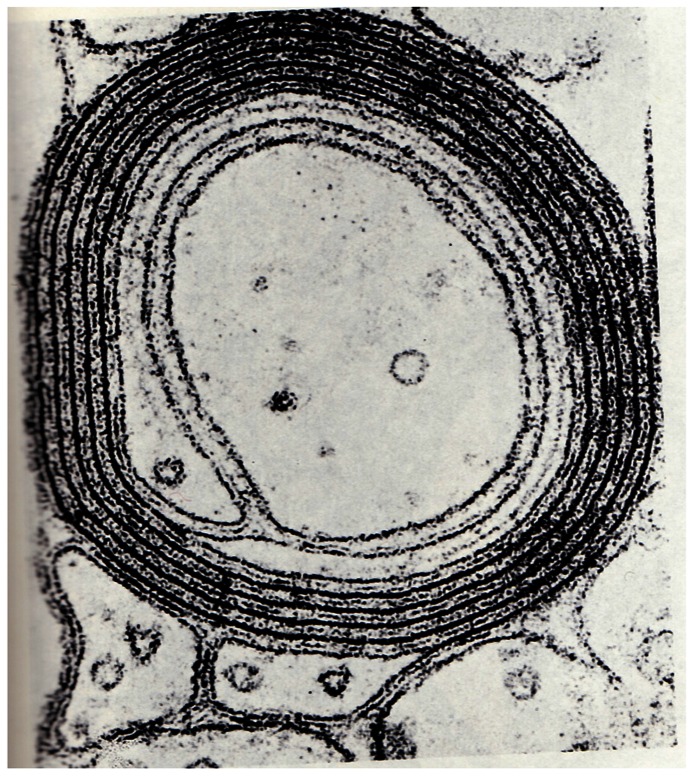
**Schwann cell**.

Finally, the myelin sheaths are the plasma membranes of oligodendrocytes and Schwann cells (**Figure 11**). This structure is also processed in the ER. The observed decline seen with age in the capacity of the ER to catalyze this processing could explain the loss of white matter consistently seen in the elderly and the slowing of thought processes as we age.

## PROPOSED CELLULAR AND ANIMAL MODELS FOR DRUG DISCOVERY

These findings would suggest that we should be seeking agents for the treatment of Alzheimer’s disease that enhance the components of the ER, posttranslational, protein processing pathway. Classically the usual procedure for such a search has been to construct test systems in which the promoter region for the target protein is linked to a reporter system, such as luciferase, a beta galactosidase or a fluorescent protein. Yet, we now know that non-coding regions of the DNA and histone modifications play major roles in controlling both transcription and translation of proteins. Hence, potentially the most fruitful approach for high-throughput drug screening would be to develop cell models in which compact fluorescent proteins, such as green fluorescent protein (GFP), are embedded in an exon of the otherwise intact target gene. This should maximize the number of potential target sites for small molecule, drug discovery.

Such transgenic models are created in embryonic or induced pluripotent stem cells by homologous recombination using a variety of site specific endonuclease systems. These models are usually created with either mouse or human embryonic or induced pluripotent stem cells. In the case of the mouse lines it would also be possible to clone the cells and create transgenic, adult animals. This is an important step since it is necessary to demonstrate that the insertion of the fluorescent protein does not lead to an animal with compromised metabolism. Usually the indicator system is inserted in either the first or last exon of the native protein. If these turn out to produce metabolically compromised cells, then it would be necessary to insert the indicator in an exon that does not compromise metabolic function. The stem cells can then be transformed into a variety of tissue types by incubating with the appropriate growth factors. With the construction of the labeled cell lines, they can be used to automatically screen large libraries of compounds in microtiter plate readers.

Similarly intact animals models could be developed in which the relevant proteins have been knocked down. It is necessary to only knock them down because knockouts of the chaperones and components of the *N*-glycosylation pathway are lethal mutants. The possible approaches could include a lox-cre system in which the specific tissue cell type, such as neuronal cells have a haploid knock out of the critical gene. Alternatively, it is possible to develop models with a global knock down of the target gene(s) with synthetic, antisense oligonucleotides.

**FIGURE 12:**
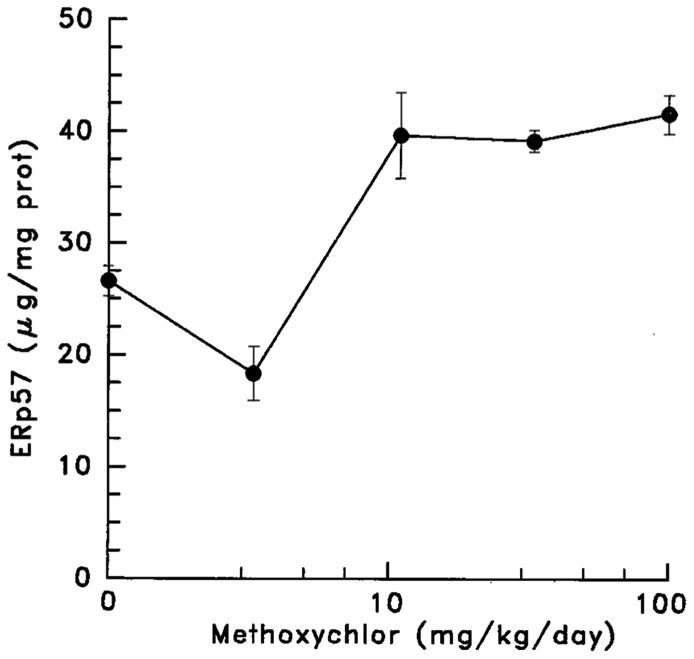
**The effect of feeding of methoxychlor for 3 weeks on the hepatic level of ERp57 (data taken from Morrell et al., 2000)**.

In an unrelated study of the toxicity of methoxychlor, we observed that feeding this insecticide to rats for 3 weeks specifically increased the levels of ERp57 in hepatic microsomes (ER) (**Figure 12**).

## CONCLUSION

1. Nine phase III clinical trials of agents that were effective in transgenic mice were not effective in patients with late onset dementia.
2. The reason that plaque does not form in the young is because Aβ is normally *N*-glycosylated and bound to two ER chaperones, ERp57 and calreticulin, which keep it in solution.
3. These components decline with age.
4. These findings suggest that the dementia seen in late onset Alzheimer’s disease is due to a decline in the ER, posttranslational processing of the synaptic, membrane proteins that are necessary for a functioning memory.
5. It also suggests that the loss of mitochondrial function and white matter in the elderly is due to this decline in ER function.
6. This paradigm suggests new cellular models for drug discovery in which fluorescent proteins are inserted into an exon of the various components of the ER, posttranslational, protein processing pathway.
7. Animal models could be constructed in which the critical proteins are knocked down by standard procedures and then candidate drugs are administered to test whether they improve cognition.

## IMPLICATIONS FOR FUTURE DRUG DISCOVERY TO TREAT ALZHEIMER’S DISEASE AND OTHER DISEASES ASSOCIATED WITH AGING

Finally, over the past two decades the pharmaceutical industry and funding agencies and foundations have made and are continuing to make massive investments in the search for disease modifying treatments for Alzheimer’s disease and other diseases of the elderly. Although these efforts have taken a variety of approaches, the bulk have sought treatments based on the Aβ hypothesis that seek to improve cognitive function by decreasing the brain content of Aβ. Considering the number of these agents currently in the pipeline, it is clear that this approach will continue to dominate drug development for the foreseeable future. Yet, since many of these new agents are merely variations on earlier failed investigational drugs, there is a significant probability that they too will not prove to be efficacious in phase III clinical trials.

In view of the long, lag period between the initial identification of new targets and the final regulatory approval of agents developed on the basis of new paradigms, it would seem prudent at this point to consider other models for drug discovery in case the current batch of investigational drugs fail to fulfill their early promise. At the present time there is little chance that this lag period can be foreshortened by evaluating therapeutic efficacy based on surrogate measures, because changes in the levels of such measures have not been demonstrated to correlate with clinical efficacy. For example, the two most widely accepted biomarkers in the CSF of disease status are low levels of Aβ and high levels of P-*tau*. Yet in the recent failed bapineuzumab trial, P-*tau* levels did decline, but the patients still showed no clinical benefit (Blennow et al., 2012).

Many have cited the experience in the treatment of hypertension in which demonstrating that decreasing blood pressure was sufficient grounds for drug approval. But this surrogate measure was based on clinical trial data that had shown that a decrease in blood pressure led to a pronounced decrease in morbid events (Veterans Administration Cooperative Study Group on Antihypertensive Agents, 1967,^1970^). In fact the decrease was so pronounced that in the present era of drug trials, a Data and Safety Monitoring Board would certainly have ended the 1970 trial no later than 3 years instead of continuing it for the full 5 years laid out in the original protocol. Until such robust surrogate measures have been identified or some agent does appear to show efficacy in phase III clinical trials which could thereby represent a template for future drug development, the most conservative approach would appear to be for industry to cast a wide net of potential targets for drug development rather than focusing on a single, possible culprit which has failed to lead to the development of effective agents.

## Conflict of Interest Statement

The author declares that the research was conducted in the absence of any commercial or financial relationships that could be construed as a potential conflict of interest.
